# Getting it right in prime time: tools and strategies for media interaction.

**DOI:** 10.3201/eid0604.000422

**Published:** 2000

**Authors:** R J Howard

**Affiliations:** Centers for Disease Control and Prevention, Atlanta, Georgia, USA.


### Getting It Right in Prime Time: Tools and Strategies for Media Interaction

"I only know what I read in the papers."
Mark Twain

Increasingly scientists are faced with the challenge of communicating with a public that may well have little understanding (or considerable misunderstanding) of their work. Bold headlines all over the world scream out urgent new health emergencies, from necrotizing fasciitis (Killer Bug Ate My Face) to avian influenza (Chicken Flu). When the popular media seek answers and information for the public, a communications strategy that uses the concept of message development and delivers timely and accurate information is very effective.

Both reporters and the public have begun to ask probing questions: Why should physicians in the United States be concerned about an outbreak of Ebola in Zaire? Why is the risk for *Escherichia coli* O157:H7 infection higher when eating undercooked hamburger than when eating undercooked steak? Should we lose sleep over West Nile virus infection? It is incumbent on the public health community to provide readily understood answers and make the communications leap from medical science to public concern. Popular access to science provided by the media has understandably created more questions.

Flow of information to the media can be facilitated by the "single overriding communication objectives" (SOCOs) approach. Use of this strategy early in the development of communication objectives streamlines data and focuses on the primary audiences. All concerned know what the message is, who the audience is, and who is going to deliver the message. This harmony is achieved by having investigators, collaborators, administrators, communications personnel, and key agency officials answer the following questions: 1) What is the key point of this interview?—Your statement should reflect what you would like to see as the lead paragraph in a newspaper story or broadcast news report about this subject. 2) What are the three facts or statistics you would like the public to remember after reading or hearing about this story? 3) Who is the main audience or population segment you would like this story to reach? Is there a secondary audience? 4) What is the single message your audience needs to take away from this report? 5) Who in your department will serve as the primary point of contact with the media and when will this person be available?

These questions are at the core of translating scientific data into useful and direct messages for the public. The process requires that the investigator scan the entire empirical structure of available data for what needs to be at the top of the data pyramid for use by the consumer. The limited time that the media will devote to this single issue must be used to deliver the most powerful message. This process ensures a uniform and effective message. For example, *E. coli* O157:H7 is a complex pathogen whose proliferation is tied to issues as far-reaching as meat production and processing, day-care centers, cooking times, handwashing, and pasteurization. But the message for the public may be as simple as "Cook hamburger until well done, drink pasteurized beverages, and wash your hands well and frequently."

If we liken the experience of being interviewed by a television or newspaper reporter to diving into a pool of water, we can see the challenge. Think of the pool of water as the data pool, and the leap into that body of water (or data) as the response to a question. The persons interviewed must decide how deep into the data they must dive. So much of scientific training dictates meticulous description of methods, a discussion of findings, assessment of validity, and statement of conclusions. But when the message is delivered to the public, communication must address the public's concerns not the scientist's.

The challenge in developing a communications strategy to deal with evolving and complex issues of public and media interest is to create a mind-set where the communicator and the institution understand the value of information exchange and can develop single overriding communication objectives for both the short-term and long-term communication goals. As an issue evolves so may the communication objectives. The initial message may be one of a warning or an advisory alerting the public to a threat. Subsequent communications may direct the public on what actions to take regarding prevention and control. Communication objectives evolve quickly and require frequent and careful development that tracks the progression of the scientific findings. The process has proven valuable in both short- and long-term communication programs. In the short term, it allows focusing on clear useful messages for the public, as was the case during the hantavirus outbreak in the southwestern United States, where residents were given simple timely health advice: "Avoid contact with rodents; don't provide havens for rodents; and report all hanta-like symptoms to your doctor immediately." In the long term, the communication process places diseases in proper perspective. Even though human cases of Ebola virus infection had not reached the shores of the United States, a global village message stressed that whether it is Ebola or West Nile virus, what happens in Zaire or the Sudan today may well be a problem in the United States tomorrow. "We live in a global village" and "diseases are only a plane flight away" are messages that everyone can understand.

**Robert J. Howard**
Centers for Disease Control and Prevention,
Atlanta, Georgia, USA

